# Exercise capacity in patients with cystic fibrosis vs. non-cystic fibrosis bronchiectasis

**DOI:** 10.1371/journal.pone.0217491

**Published:** 2019-06-13

**Authors:** Ronen Bar-Yoseph, Anat Ilivitzki, Dan M. Cooper, Michal Gur, Gur Mainzer, Fahed Hakim, Galit Livnat, Zeev Schnapp, George Shalloufeh, Merav Zucker-Toledano, Yael Subar, Lea Bentur

**Affiliations:** 1 Pediatric Pulmonary Institute, Ruth Children’s Hospital, Rambam Health Care Campus, Haifa, Israel; 2 Pediatric Radiology unit, Ruth Children’s Hospital, Rambam Health Care Campus, Haifa, Israel; 3 The Bruce Rappaport Faculty of Medicine, Technion–Israel Institute of Technology, Haifa, Israel; 4 Pediatric Exercise and Genomics Research Center (PERC), Department of Pediatrics, University of California Irvine, Irvine, CA, United States of America; 5 University of California Irvine Institute for Clinical and Translational Science, Irvine, CA, United States of America; 6 Pediatric Cardiology, The Baruch Padeh Medical Center, Poriya, Israel; 7 Pediatric pulmonology unit, Carmel Medical Center, Haifa, Israel; 8 Department of Pediatrics, Carmel Medical Center, Haifa, Israel; 9 Department of Pediatrics A, Ruth Children’s Hospital, Rambam Health Care Campus, Haifa, Israel; 10 Pediatric Cardiology, Ruth Children’s Hospital, Rambam Health Care Campus, Haifa, Israel; Akershus University Hospital, NORWAY

## Abstract

**Background:**

Bronchiectasis is associated with morbidity, low exercise capacity and poor quality of life. There is a paucity of data on exercise capacity using cardiopulmonary exercise test (CPET) in non-cystic fibrosis (CF) bronchiectasis. Our aim was to compare exercise capacity using CPET in CF and non-CF bronchiectasis patients.

**Methods:**

Cross-sectional retrospective/prospective controlled study assessing CPET using cycle ergometer. Exercise parameters and computed tomography (CT) findings were compared. Results: Hundred two patients with bronchiectasis and 88 controls were evaluated; 49 CF (age 19.7 ± 9.7 y/o, FEV_1_%predicted 70.9 ± 20.5%) and 53 non-CF (18.6 ± 10.6 y/o, FEV_1_%predicted 68.7 ± 21.5%). Peak oxygen uptake (peak ${\dot{V}O}_{2}$) was similar and relatively preserved in both groups (CF 1915.5±702.0; non-CF 1740±568; control 2111.0±748.3 mL/min). Breathing limitation was found in the two groups vs. control; low breathing reserve (49% in CF; 43% non-CF; 5% control) and increased $\dot{V}E/\dot{V}CO_{2}$ (CF 31.4±4.1, non-CF 31.7±4.1 and control 27.2 ± 2.8). Oxygen pulse was lower in the non-CF; whereas a linear relationship between peak ${\dot{V}O}_{2}$ vs. FEV_1_ and vs. FVC was found only for CF. CT score correlated with $\dot{V}E/\dot{V}CO_{2}$ and negatively correlated with ${\dot{V}O}_{2}/\mathrm{kg}$ and post exercise oxygen saturation (SpO_2_).

**Conclusions:**

CPET parameters may differ between CF and non-CF bronchiectasis. However, normal exercise capacity may be found unrelated to the etiology of the bronchiectasis. Anatomical changes in CT are associated with functional finding of increased $\dot{V}E/\dot{V}CO_{2}$ and decreased SpO_2_. Larger longitudinal studies including cardiac assessment are needed to better study exercise capacity in different etiologies of non-CF bronchiectasis.

**Trial registration:**

ClinicalTrials.gov, registration number: NCT03147651.

## Introduction

Bronchiectasis is associated with considerable morbidity and poor quality of life [1,2]. Often, a distinction is made between bronchiectasis caused by cystic fibrosis (CF) and non-CF bronchiectasis. The etiology for non-CF bronchiectasis include primary ciliary dyskinesia (PCD), post infectious, aspiration, primary and secondary immunodeficiency, congenital malformation, idiopathic and others [3]. While management and follow up strategies for PCD are usually extrapolated from CF, for the other non-CF bronchiectasis etiologies data is scarce.

In the last several decades, exercise has been encouraged for CF patients to improve sputum expectoration, lung function and quality of life and to decrease morbidity and mortality [4,5]. Patients with bronchiectasis show reduced daily habitual physical activity and exercise capacity [6–8]. This may be due to respiratory limitation (assessed by CPET) leading to decreased exercise tolerance, peripheral muscular pathophysiology or due to secondary factors such as deconditioning or overly cautious caregivers restraining patient’s physical exertion [7,9]. CF patients often have multi organ disease that could involve nutritional deficits and specific muscle dysfunction compared to other common bronchiectasis etiologies (e.g. PCD) [10]. Heart involvement was shown in children and adult CF patients with correlation to pulmonary function [11]. Cardiopulmonary exercise testing (CPET) is increasingly gaining importance in clinical medicine and provides clinical insight into overall fitness, exercise limitations, disease prognosis, and management through exercise based therapeutic interventions [12]. CPET was recently used to assess treatment response of mutation specific therapy and gene therapy in CF patients [13]. Unlike CPET in CF, there is a paucity of data on exercise capacity in non-CF bronchiectasis patients.

The primary purpose of the present study was to assess exercise capacity in CF and non-CF bronchiectasis patients. In addition, we explored the relationship between CPET results and anatomical changes in CT findings. This may expand the scope of using CPET outcome indices to assess, monitor and predict disease progression and response to treatment.

## Methods

This study was conducted in accordance with the amended Declaration of Helsinki. The study was approved by the Helsinki Committee (Institutional Review Board) of Rambam Health Care Campus (application number 0048-15-RMB), and Written consent was obtained from patients over the age of 18 years or from parents of minors prior to initiating CPET in the prospective part. This was a cross-sectional retrospective (2013–2016)/prospective (Since 2017) study population. The study was performed during a single visit in a CPET lab, situated in a tertiary university-affiliated medical center. The retrospective study included data analysis of patients who performed exercise tests as part of their clinical evaluation. Inclusion criteria for both groups were: evidence of bronchiectasis by CT, age > seven years, height > 125cm, and completed a maximal CPET test according to the accepted criteria. Exclusion criteria were preforming submaximal CPET, lack of data from the exercise test, exacerbation of patient’s condition within 30 days before the exercise evaluation, other chronic diseases affecting test results and according to the American College of Sports Medicine (ACSM) guidelines [14]. Control group was consisted of age and sex matched healthy subjects previously recruited as control for other studies in our lab or healthy subjects who were referred to our institute with a minor exercise complain and found to have no exercise limitations.

### Anthropometric and disease measures

Age, sex, height, body mass, body mass index (BMI) as well as z-scores for body mass index (BMI) based on Center of disease and control (CDC) criteria. Etiology of bronchiectasis, chest CT and echocardiography were recorded. Each patient completed a questionnaire regarding recent illness, medication use, lifestyle and exercise capacity in daily life. Baseline pre-CPET pulmonary function was used for analysis of potential breathing limitation during exercise.

### Spirometry

Spirometry was performed before and after CPET in accordance with the European Respiratory Society and American Thoracic Society guidelines using a Quark PFT spirometer (Cosmed, Italy). Spirometry measured subjects forced vital capacity (FVC), forced expiratory volume in one second (FEV_1_), and maximum voluntary ventilation (MVV) was calculated using the 12-second sprint method.

### Exercise testing with CPET

CPET was carried out by an experienced technician and/or exercise physiologist using a Quark CPET metabolic cart (Cosmed, Rome, Italy) according to the ATS guidelines [15]. Cycle ergometer progressive exercise testing was performed to the limit of the participant’s tolerance. Cycling began with a zero resistance warm up lasting two-three minutes and followed with an incrementing resistance (10–25 watts/minute) that was adapted to the patient’s functional capacities (ramp protocol) up to exhaustion. Subjects were asked to maintain a pedal speed at the desired protocol level, 60–65 rpm. Gas exchange variables through a designated face mask (V2 mask, Cosmed, Rome, Italy), 12-lead ECG, blood pressure and oxygen saturation (SpO_2_) were recorded at rest, during the test and during the recovery period. SpO_2_ was measured continuously using Masimo SET 2000 (Schiller) and recorded at baseline, every 120 seconds, peak exercise and one, two- and five-minutes post exercise.

Criteria for terminating the test were inability to maintain pedaling cadence (<60 rpm), in association with subjective evidence of fatigue (sweating, hyperpnea), and one or more of the following: peak ${\dot{V}O}_{2}$ > 80% predicted, maximal heart rate > 80% HR predicted (HRpred = 208- (AgeX0.7)) [16], RER > 1.0 for age< 18 years and RER > 1.05 for adults, or reaching a ${\dot{V}O}_{2}$ plateau (failure to increase oxygen uptake despite a continues increase in work). Breathing reserve (BR) was calculated as [MVV-peakVE]/ MVV and low breathing reserve (BR) defined as BR %< 15% or BR<11 liter/min [17,18].

### CT Scoring

CT scans were performed every three years as part of the routine follow-up of our patients and were evaluated (Bhalla score) by a trained radiologist. The Bhalla score and the modified Bhalla score has been previously used in CF and non-CF bronchiectasis in children and adults [19–24]. The score includes the extent of bronchiectasis and number of segments involved, peribronchial thickening, mucus plugs, sacculations and abscesses, bullae, emphysema, and collapse and consolidation. The total score ranges from 0–25, with a higher score indicating more severe changes.

### Statistical analysis

This was a retrospective-prospective controlled study. Descriptive statistics presented in the form of mean ± standard deviation, median [25–75 percentiles], percentage and Z score, as applicable. The independent variables for this analysis include subject condition (CF bronchiectasis and non-CF bronchiectasis) and the acute bout of exercise test. The primary outcome parameter was the peak oxygen uptake. Secondary outcome parameters were spirometry, vital signs (heart rate (HR) and oxygen saturation (SpO_2_)), oxygen pulse (O_2_ pulse, ${\dot{V}O}_{2}/\mathrm{HR}$), $\dot{V}E/\dot{V}CO_{2}$ and CT scores. All data was analyzed using SPSS version 21.0 (IBM, SPSS Chicago, Illinois). Statistical significance was set *a priori* at an alpha-level of p ≤ 0.05. Descriptive statistics in terms of exercise capacity differences between the two study groups were analyzed using a non-paired t-test. Quantitative parameters that are not normally distributed were analyzed by the Mann-Whitney U test. Fisher exact test was used for differences in the categorical parameters. Finally, a Pearson correlation was used to determine the relationship between peak ${\dot{V}O}_{2}$ and FVC or FEV_1_ and relationship between CT scores and pulmonary and exercise parameters.

## Results

A total of one hundred and two patients (49 CF and 53 non-CF bronchiectasis patients) and 88 age and sex matched controls were included in the data analysis (Table 1). No adverse events were recorded. Non-CF bronchiectasis etiologies is summarized in Table 2. Two subjects in the control group did not perform spirometry due to technical issues.

**Table 1:** Patient’s demographic and anthropometrics.

	CF (n = 49)	Non-CF (n = 53)	Control (n = 88)	*p*
**Age** (yrs)	19.7 ± 9.7	18.6 ± 10.6	19.9±11.9	NS
**Gender–Male** (n, (%))	31 (63.0%)	32 (60%)	46 (52%)	NS
**Height** (cm)	157.6 ± 16.9	156.1 ± 16.3	158.4±17.2	NS
**Weight** (kg)	53.9 ± 18.9	50.8 ± 18.2	55.2±20.2	NS
**BMI**	21.1 ± 4.4	20.4 ± 5.0	20.9±4.2	NS
**BMI Z-score**	0.19 [(-0.72)—(0.85)]	-0.33 [(-1.54)—(0.68)]	0.32 [(-0.41)—(1.09)]	NS

^1^Control vs CF

^2^Control vs Non-CF

CF–cystic fibrosis, BMI—body mass index; BMI Z-score of the adults population was adjusted to age 19 years.

Results are expressed as mean ± standard deviation, median [25–75 percentiles], percentage and Z score, as applicable.

**Table 2:** Non-CF bronchiectasis etiologies.

Etiology	N
Idiopathic	14
Primary Ciliary Dyskinesia (PCD)	14
Post Infectious bronchiectasis	7
Bronchiolitis Obliterans (BO) induced bronchiectasis	5
Tracheoesophageal fistula (TEF)	4
Bronchopulmonary dysplasia (BPD)	3
Others	6
**Total**	**53**

Cardiorespiratory parameters are summarized in **Table 3**. Pulmonary function tests were mildly reduced with no difference between groups. Peak oxygen uptake was not statistically different in both groups and only for the non-CF group was lower than the control group (p = 0.007). Pre-exercise and peak SpO_2_ were preserved and similar between groups. A statistically significant SpO_2_ deterioration between baseline and peak exercise was observed in both groups (1% for both groups, p = 0.026 for CF, p = 0.024 for non-CF).

**Table 3:** Cardiopulmonary parameters in CF and Non-CF bronchiectasis patients.

	CF (n = 49)	Non-CF (n = 53)	Control (n = 88)	*p* value		
				Control vs CF	Control vs Non-CF	CF vs Non-CF
**FEV**_**1**_ (L/Sec) *	2.1 ± 0.83	2.0 ±0.9	2.9 ± 1.03	<0.0001	<0.0001	NS
**FEV**_**1**_ (% Predicted) *	70.9 ± 20.5	68.7 ± 21.5	99.1 ± 12.4	<0.0001	<0.0001	NS
**FVC** (L) *	2.8 ± 1.0	2.7 ±1.1	3.5 ± 1.3	<0.005	<0.005	NS
**FVC** (% Pred) *	82.9 ± 18.5	79.9 ± 20.6	102.2 ± 12.0	<0.0001	<0.0001	NS
**peak** ${\dot{V}O}_{2}$ (mL/min)	1915.5 ± 702.0	1740 ± 568	2111.0 ± 748.3	NS	0.007	NS
**peak** ${\dot{V}O}_{2}$ (%Pred)	92.9 ± 21.9	87.7 ± 19.0	101.6 ± 19.7	0.049	<0.0001	NS
**peak** ${\dot{V}O}_{2}/\mathrm{kg}$ (mL/kg/min)	37.7 ± 10.3	35.3 ± 10.8	39.6 ± 8.9	NS	0.035	NS
**RER**	1.05 [0.98–1.13]	1.03 [0.98–1.10]	1.13 [1.03–1.20]	<0.01	<0.01	NS
**Peak HR (beats/min)**	180 [167–192[	182 [172–190]	182 [175–191]	NS	NS	NS
**Peak HR (%pred)**	89 [85–96]	92[87–96]	94 [92–97]	0.001	NS	NS
**Lowest** $\dot{V}E/\dot{V}CO_{2}$	31.4 ± 4.1	31.7 ± 4.1	27.2 ± 2.8	<0.0001	0.008	NS
${\dot{V}O}_{2}/\mathrm{peakHR}$ (mL/min/beat)	10.8 ± 3.9	9.6 ± 3.0	11.6 ± 4.3	NS	0.010	NS
${\dot{V}O}_{2}/\mathrm{peakHR}$ (%Pred)	100.6 ± 21.8	92.6 ± 18.3	108.0 ± 20.5	NS	<0.0001	0.046
$peak\dot{V}E$ (L/min)	68.8 ± 27.4	60.2 ± 22.7	77.3 ± 31.1	NS	0.002	NS
**MVV** (L/min)	86.1 ± 35.4	81.6 ± 35.9	120.8 ± 42.9	<0.0001	<0.0001	NS
**SpO**_**2**_ **(%)** (pre)	98.3 ± 1.8**	98.7 ± 2.3***	99.5 ± 0.86	0.001	0.032	NS
**SpO**_**2** at peakVO2_ **(%)** (post)	97.4 ± 4.0**	97.7 ± 4.9***	99.3 ± 0.99	0.006	0.023	NS
**Low Breathing Reserve** n (%)	24 (49%)	2**3** (4**3**%)	4 (5%)	<0.0001	<0.0001	NS
**CT score**	9.23±5.9	9.10±5.1		NA	NA	NS

CF–cystic fibrosis, FEV_1_-forced expiratory volume in one second, FVC- forced vital capacity, ${\dot{V}O}_{2}$-oxygen uptake, RER—respiratory exchange ratio, $\dot{V}E$-minute ventilation, $\dot{V}CO_{2}$-carbon dioxide production, HR- heart rate, MVV- maximum voluntary ventilation, SpO_2_ –oxygen saturation.

* n = 86 for the control group

** SpO_2_ pre vs. post in CF: p = 0.026

*** SpO_2_ pre vs. post in non- CF: p = 0.024

Results are expressed as mean ± standard deviation, median [25–75 percentiles], percentage and Z score, as applicable.

Low breathing reserve was observed in 49% of CF patients and 43% of non-CF patients with no statistically difference between the groups and statistically higher (p<0.0001) vs. control (5%). Ventilatory equivalent for $\dot{V}CO_{2}$ (lowest $\dot{V}E/\dot{V}CO_{2}$ measured during ramp protocol) was elevated compared to controls (p<0.01) and similar between groups.

Peak O_2_ pulse (${\dot{V}O}_{2}/\mathrm{HR}$) was not statistically different between the two groups. However, O_2_ pulse (%predicted) was higher in the CF group than in the non-CF group. Both values were in the normal range, and only for the non-CF group was lower than the control group.

Echocardiography records were available for 54 patients (17 CF, 37 non-CF) and were re-evaluated by a pediatric cardiologist. Seven non-CF patients had a structural abnormality (six with situs inversus and one post coarctation of aorta repair), compared to one CF patient (patent foramen ovale). Other findings in the non-CF group were three patients with mild mitral valve insufficiency, three with mild pulmonary hypertension, one with mild aortic and pulmonary valves regurgitation and one with mild tricuspid regurgitation were observed. In the CF group, one patient had mild mitral valve prolapse.

CT scans were available for evaluation in 44 CF and 48 non-CF bronchiectasis patients. The CT Bhalla score was similar between the groups. CT scores were correlated with $\dot{V}E/VCO_{2}$ and inversely correlated with FVC %pred, FEV_1_%pred, peak ${\dot{V}O}_{2}/\mathrm{kg}$, and SpO_2_ (post) for the whole group of patients. Similar inverse correlations between CT scores and FEV_1_%pred and SpO_2_ post were found for both groups. CT scores were also inversely correlated with FVC % and MVV only in CF patients. The correlation coefficients between CT and parameters of spirometry and exercise in bronchiectasis patients are presented in the Supplement (S1 Table).

**Fig 1:**
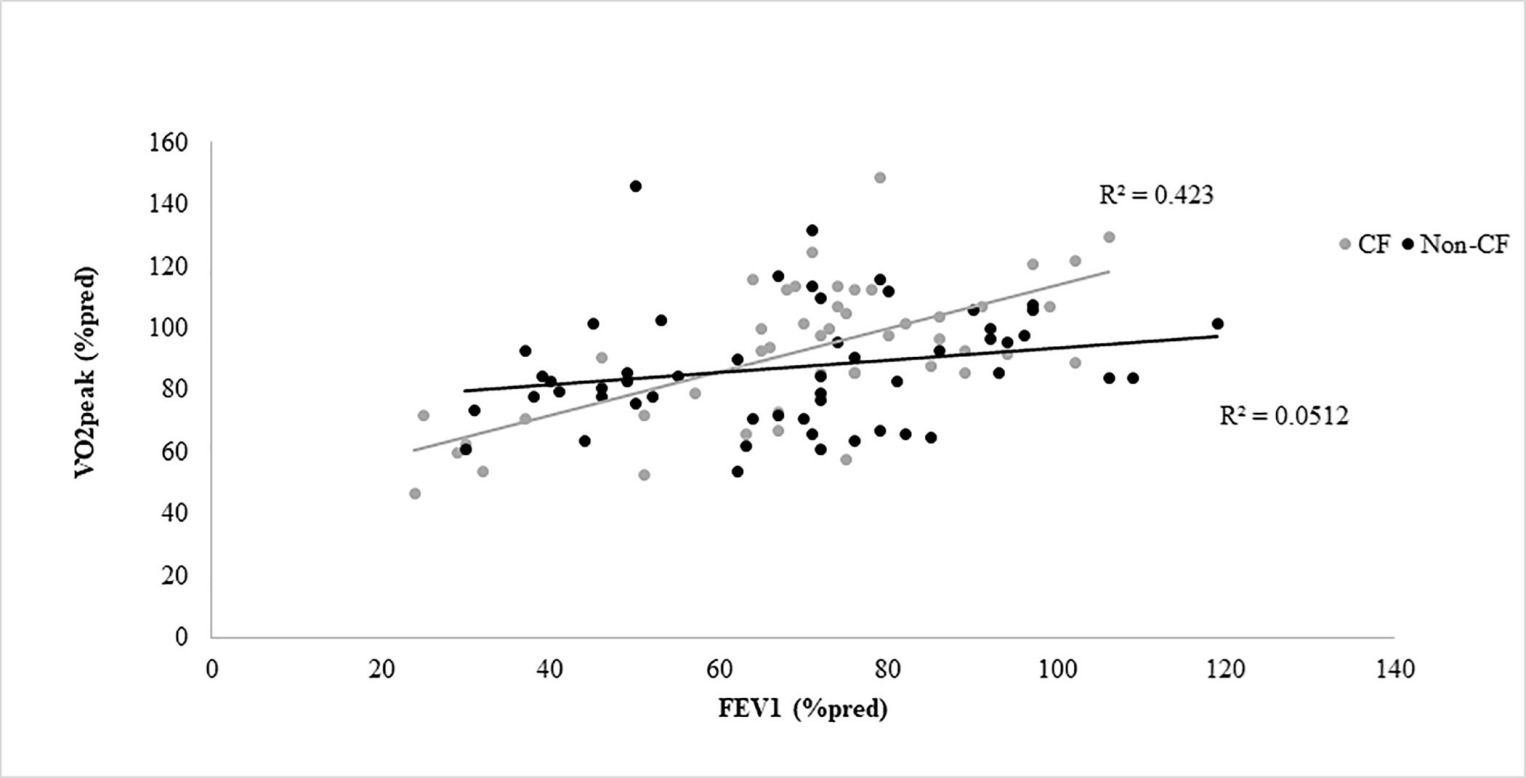
Baseline FEV1 (%pred) and peak ${\dot{V}O}_{2}$ (%pred) relationship. Moderate linear relationship (r = 0.63, p<0.0001) is demonstrated for cystic fibrosis (CF) patients (gray circles) while weak linear relationship (r = 0.23, p = 0.1) for the non-CF patients (black circles).

Pearson correlation was used to determine the relationship between peak ${\dot{V}O}_{2}$ and FEV_1_ or FVC and a positive correlation was found only in the CF group (Fig 1 and S1 Fig).

## Discussion

This cross-sectional retrospective/prospective controlled study evaluated CPET in a large CF and non-CF bronchiectasis cohort and compared the results to a control group. Peak ${\dot{V}O}_{2}$ was relatively preserved while both groups had breathing limitation (low breathing reserve and elevated $\dot{V}E/\dot{V}CO_{2}$). Oxygen pulse%pred was lower compared to CF and control; whereas a linear relationship between peak ${\dot{V}O}_{2}$ and FEV_1_ and peak ${\dot{V}O}_{2}$ vs. FVC was found only for CF patients. CT score correlated with $\dot{V}E/\dot{V}CO_{2}$ and negatively correlated with ${\dot{V}O}_{2}/\mathrm{kg}$ and post exercise SpO_2_.

The two groups (CF and non-CF bronchiectasis) have similarities and dissimilarities. In term of pathophysiology, the CF group is characterized by abnormal CFTR whereas different pathophysiology underlines the non-CF bronchiectasis. While there are guidelines for CF management and follow up, strategies for PCD (extrapolated from CF) are less established, and for the other non-CF bronchiectasis treatment data is scarce. The end result in the two groups is structural bronchiectasis but, the distribution, rate of progression, the degree of inflammation and type of infection may differ between these groups. CF patients often have multi organ disease (especially pancreatic insufficiency) that could lead to primary nutritional deficits and specific muscle dysfunction; while in the non-CF group nutritional deficits may be secondary to lung disease [10].

In term of heart; abnormal CFTR expression is found in the CF myocardium [25], Recently, the concept of early CF related cardiomyopathy has been suggested and include functional and inflammatory mechanisms [11,26]. However, the precise physiological role of abnormal CFTR is not fully understood. Non-CF patients (e.g. PCD) may have structural abnormalities that theoretically can affect cardiac function.

To the best of our knowledge, only two groups compared exercise capacity in CF and non-CF bronchiectasis patients in relatively small study population. Edwards et al (18 CF and 18 non-CF) and Stevens et al (19 CF, 8 non-CF) compared exercise capacity in pediatric patients to healthy controls. No statistical differences could be demonstrated between study groups; peak oxygen uptake was reduced for both groups compared to healthy controls [7,21,27]. Relative and similarly preserved peak ${\dot{V}O}_{2}$ was found in our study groups. While most of the studies reported reduced exercise capacity in CF patients, some found normal values, mainly in pediatric patients with relatively preserved pulmonary function [9,10,28,29]. In our group of mild-moderate CF patients, peak ${\dot{V}O}_{2}$ was preserved potentially due to the relatively young cohort and disease severity.

Few studies assessed exercise capacity in non-CF bronchiectasis. Similar to other studies, idiopathic and PCD were the most common etiologies in our group of non-CF bronchiectasis [3,7,30]. Swaminathan et al. reported reduced exercise capacity in 17 idiopathic bronchiectasis pediatric patients (mean FEV_1_ = 80%pred) compared to healthy controls [6]. Studies evaluating exercise capacity in young PCD patients are contradictory. Adolescent PCD patients had preserved and similar exercise capacity compared to CF patients and healthy controls [10], while a reduced exercise capacity was demonstrated in other studies [31,32]. Herein, lower peak ${\dot{V}O}_{2}$ was observed in our non-CF group compared to control and may reflect different disease pathophysiology and inclusions of different subgroups.

A moderate relationship between ${\dot{V}O}_{2}$ peak and FEV_1_ and FVC was found only in CF patients. This relationship in CF was previously described [9,33] and is likely due to the fact that CF was a relative homogenous group in term of etiology and standard of care. In contrast, the non-CF bronchiectasis group includes different etiologies that may present different relationship between exercise capacity and pulmonary function. In PCD patients aerobic fitness correlated with FEV_1_ [32] while for other etiologies of non-CF bronchiectasis there is still scarce data regarding these relationships.

We calculated breathing reserve. The practice of using MVV is equivocal with no one uniform practice to be followed. Measuring flow-volume loops obtained during exercise and plotting them according to a measured end-expiratory lung volume within the maximal flow-volume, has been suggested to provide more information on the sources and degree of ventilatory constraint [34]. Flow-volume loops during exercise were not performed in our study. Recent multicenter study from the CFTR exercise group use similar approach for evaluating breathing reserve [35]. As expected, we found high incidence of respiratory limitation, represented as low BR and high ventilatory equivalents ($\dot{V}E/\dot{V}CO_{2}$), in both patient groups, which was statistically different from the control group. This may be due to abnormal ventilatory control, ventilation inhomogeneity and hyperinflation [6,9,31,32] and in CF patients was found to correlate with mortality [28].

Decreased oxygen delivery to the working muscles during exercise potentially can affect exercise capacity and can be demonstrated by oxygen desaturation and decreased O_2_ pulse. A small but statistical decrease in SpO_2_ was found for both groups pre vs. peak-exercise. When assessing SpO_2_ indirectly (e.g. pulse oximeter) without an arterial sample to assess PaO_2_ relatively small changes in the normal range of SpO_2_ are a physiological challenge. Theoretically decreased SpO_2_ combined with high $\dot{V}E/\dot{V}CO_{2}$ may suggests impaired alveolar ventilation. However, as these changes were in the flat, upper portion of oxyhemoglobin dissociation curve, their clinical significance is of minor relevance.

Mean O_2_ pulse indices were preserved in both groups, with lower absolute values compared to control only for the non-CF. Lower O_2_ pulse%pred was found in the non-CF compared to CF and control groups. O_2_ pulse can be a marker for decreased cardiac stroke volume.

These different patterns observed between the two groups may reflect the differences in etiology between study groups. In our study, abnormal echocardiographic findings were more common in the non-CF group and could have a potential effect on CPET results.

Similar CT Bhalla scores were found in the CF and non-CF patients. There was a good correlation between CT and submaximal exercise parameters ($\dot{V}E/\dot{V}CO_{2}$) and inverse correlation with peak exercise parameters (${\dot{V}O}_{2}/\mathrm{kg}$) and spirometry (FEV_1_ and FVC). Post CPET SpO_2_ correlated with CT score in both groups, probably reflecting ventilation-perfusion mismatch and increased dead space. CT correlated with spirometry better in CF than in non-CF. To the best of our knowledge, only one group compared CT findings and exercise parameters in CF and non-CF patients. The study was limited to peak ${\dot{V}O}_{2}$ and HR and to pediatric population and no correlation was found between CT and exercise parameters or spirometry [21]. Dodd et al showed a strong correlation between CT and exercise capacity in young adult CF patients and the correlation was better than between exercise capacity and clinical measurements (FEV_1_ and BMI) [24]. We did not find any similar study in non-CF bronchiectasis. Correlation between CT and spirometry in bronchiectasis patients was previously described with contradictory results. Studies in CF found good correlation in children and young adults [36–38] and similar correlation was reported in young PCD patients [22] and for adult non-CF bronchiectasis patients [39] while other studies showed no correlation [21,38].

The main strengths of this study are the relatively large cohort for each group, the age range that covers the transition from childhood to adulthood and the radiographic correlation.

### Limitations

The non-CF group is a heterogeneous group; each subgroup included few patients. For example, two patients (out of 38 non-CF patients) had significant structural heart defects which may affect the results of peak exercise values in non-CF bronchiectasis group. The study emphasizes the heterogeneity of non-CF bronchiectasis and the need to have a larger group that would allow sub analysis of the different etiologies. Careful cardiac assessment was not done systematically for both groups and muscle limitation and habitual physical activity were not assessed.

### Conclusions

CPET parameters may differ between CF and non-CF bronchiectasis. However, normal exercise capacity may be found unrelated to the etiology of the bronchiectasis. Anatomical changes in CT are associated with functional finding of increased $VE/\dot{V}CO_{2}$ and decreased post exercise SpO_2_ assessed by CPET. Evaluating CPET variables in non-CF bronchiectasis may be used for individualized exercise prescription, monitoring disease progression and for assessing treatment strategies. Larger longitudinal studies are needed to better study exercise capacity in different etiologies of non-CF bronchiectasis.

## Supporting information

S1 FigBaseline FVC (%pred) and peak ${\dot{V}O}_{2}$ (%pred) relationship.Moderate linear relationship (r = 0.68, p<0.0001) is demonstrated for cystic fibrosis (CF) patients (open circles) while weak linear relationship (r = 0.3, p = 0.027) for the non-CF patients (grey circles).(TIF)

S1 Table Correlations of CT Bhalla score with lung function tests and exercise parameters. (DOCX)

S1 File Patients data. (PDF)

## Abbreviations

CF: Cystic fibrosis
CPET: Cardiopulmonary exercise testing
CT: computed tomography
FEV_1_: forced expiratory volume in one second
HR: heart rate
MVV: maximum voluntary ventilation
PCD: Primary ciliary dyskinesia
SpO_2_: oxygen saturation
$\dot{V}E$: minute ventilation
$\dot{V}CO_{2}$: carbon dioxide production
$\dot{V}O_{2}$: oxygen uptake
