# Supplementary figures and images for: Exercise capacity in patients with cystic fibrosis vs. non-cystic fibrosis bronchiectasis
Source: PLoS One. 2019 Jun 13;14(6):e0217491. doi: 10.1371/journal.pone.0217491 (PMC6563963; doi:10.1371/journal.pone.0217491)

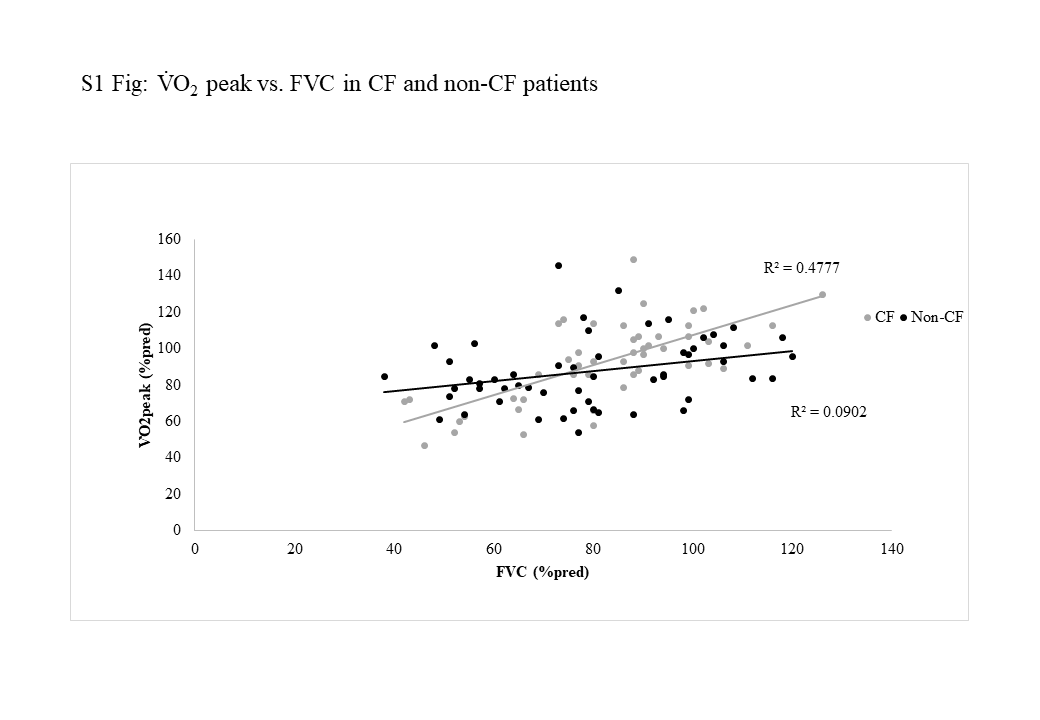

Supplement: S1 Fig — Moderate linear relationship (r = 0.68, p<0.0001) is demonstrated for cystic fibrosis (CF) patients (open circles) while weak linear relationship (r = 0.3, p = 0.027) for the non-CF patients (grey circles). (TIF) [file pone.0217491.s001.tif]
