# Supplementary material for: Exercise capacity in patients with cystic fibrosis vs. non-cystic fibrosis bronchiectasis
Source: PLoS One. 2019 Jun 13;14(6):e0217491. doi: 10.1371/journal.pone.0217491 (PMC6563963; doi:10.1371/journal.pone.0217491)
Supplement: S1 Table — (DOCX) [file pone.0217491.s002.docx]

**S1 Table**

Correlations of CT Bhalla score with lung function tests and exercise parameters

|  | Total  (n=93) | | CF  (n=44) | | Non-CF  (n=49) | |
| --- | --- | --- | --- | --- | --- | --- |
|  | r | *p* | r | *p* | r | *p* |
|  | | | | | | |
| FVC (L) | -0.021 | 0.845 | -0.253 | 0.097 | 0.206 | 0.156 |
| FVC (%pred) | -0.256 | 0.013 | -0.302 | 0.046 | -0.217 | 0.135 |
| FEV_1_ (L/Sec) | -0.140 | 0.184 | -0.348 | 0.022 | 0.061 | 0.676 |
| FEV_1_ (%pred) | -0.351 | 0.001 | -0.410 | 0.006 | -0.295 | 0.039 |
| Peak V̇O_2_ (ml/min) | -0.060 | 0.567 | -0.233 | 0.127 | 0.175 | 0.228 |
| Peak V̇O_2_ (%pred) | -0.193 | 0.064 | -0.242 | 0.114 | -0.136 | 0.350 |
| peak V̇O_2_/kg (mL/kg/min) | -0.235 | 0.023 | -0.236 | 0.123 | -0.243 | 0.093 |
| V̇E/VCO_2_ (lowest) | 0.244 | 0.018 | 0.285 | 0.060 | 0.199 | 0.171 |
| V̇O_2_/HR (mL/min/beat) | -0.004 | 0.968 | -0.193 | 0.209 | 0.271 | 0.060 |
| V̇O_2_/HR (%pred) | -0.186 | 0.075 | -0.286 | 0.059 | -0.067 | 0.646 |
| SpO_2_ Pre | -0.173 | 0.098 | -0.241 | 0.114 | -0.122 | 0.403 |
| SpO_2_ Post | -0.349 | 0.001 | -0.417 | 0.005 | -0.301 | 0.036 |
| V̇E (L/min) | -0.034 | 0.750 | -0.229 | 0.135 | 0.224 | 0.121 |
| MVV (L/min) | -0.110 | 0.294 | -0.313 | 0.038 | 0.112 | 0.442 |

CF – cystic fibrosis, FEV_1_-forced expiratory volume in one second, FVC- forced vital capacity, V̇O_2_-oxygen uptake, V̇E-minute ventilation, V̇CO_2_- carbon dioxide production, HR- heart rate, MVV- maximum voluntary ventilation, SpO_2_ – oxygen saturation.
