# Supplementary material for: Exercise capacity in patients with cystic fibrosis vs. non-cystic fibrosis bronchiectasis
Source: PLoS One. 2019 Jun 13;14(6):e0217491. doi: 10.1371/journal.pone.0217491 (PMC6563963; doi:10.1371/journal.pone.0217491)
Supplement: S1 File — (PDF) [file pone.0217491.s003.pdf]

Bronchiectasis etiology	CT score	Breathing Reserve (%)	Breathing Reserve (L)	MVV (L/min)	VE(L/min)	VO2 Specific (ml/kg/min)	Sat. O2 Post	Sat. O2 Pre	O2 pulse	O2 pul% pred	Lowest VE/VCO2	HR (%pred)	HR max	RER	VO2 (%Pred)	VO2 Absolute ml/min	FEV 1.0 (% Pred)	FEV 1.0 (L/Sec)	FVC (% Pred)	FVC (L)	BMI z score	BMI percentiles (a	BMI	WEIGHT (kg)	HEIGHT (cm)	Gender	DOB (Date of	Subject No.
CF	4	8.3%	4	48	44	47.4	100	100	6.9	126	32	87%	185	1.03	114	1281	69	1.2	73	1.43	0.26	60%	16	27	130	M	14/08/2007	1
CF	12	27.1%	26.8	98.8	72	34.43	99	99	11.3	107	26.8	99%	192	1.02	105	2169	75	2.47	88	3.28	0.66	75%	24.3	63	161	F	26/07/1995	2
CF	4	17.1%	14.2	83.2	69	34.2	100	100	11.1	109	31	87%	181	0.98	102	2018	70	2.08	111	3.68	0.86	81%	21.4	59.5	166.5	F	30/08/2001	3
CF	16	9.5%	4	42	38	22	84	93	10.2	68	28	88%	158	0.93	60	1550	29	1.05	53	2.24	0.73	77%	25	69	166	M	30-02-1976	4
CF	0	48.2%	41	85	44	26.7	98	100	10.3	102	22	96%	181	1.00	98	1922	72	2.13	77	2.64	1.46	93%	29	72	157	F	11/11/1989	5
CF		20.9%	14	67	53	41	100	100	8.7	140	30	100%	198	1.00	92	1625	94	1.69	103	2.11	-0.11	46%	22	29	133	M	25/12/2003	6
CF	17	13.6%	9	66	57	42	100	100	6.9	106	37	89%	185	1.05	113	1300	78	1.49	86	1.76	-0.56	29%	16.2	30	136	F	10/12/2002	7
CF	1	20.4%	20.2	99.1	78.9	30.68	98	98	15.3	102	27.6	92%	171	1.02	94	2608	66	2.44	75	3.27	1.76	96%	30.5	85	167	M	27/07/1979	8
CF	3	24.1%	27	112	85	48.7	100	100	9.8	87	30	96%	199	1.11	107	1941	91	2.49	99	3.1	-1.15	13%	16.6	39.8	155	M	27/05/2000	9
CF	9	14.0%	8	57	49	23	98	97	10.6	75	35	93%	163	0.96	71	1728	37	1.26	42	1.77	0.92	82%	25.8	75.5	171	M	12/04/1965	10
CF	0	35.2%	43	122	79	35	100	100	10.9	74	30	103%	197	1.19	67	2000	67	3.05	65	3.49	-1.96	2%	18.2	57	177	M	17/04/1993	11
CF	19	43.3%	27.2	62.8	35.6	23.06	99	100	5.7	63	32.2	86%	167	1.13	53	950	51	1.57	66	2.31	-2.19	1%	16.9	41.2	156	F	23/11/1994	12
CF	16	-0.6%	-0.2	30.8	31	24.7	94	95	6.7	69	41	70%	152	0.9	54	1013	32	0.77	52	1.43	-0.64	26%	19	41.6	148	M	18/07/1998	13
CF	12	22.1%	21.1	95.6	74.5	40.83	98	97	14.9	135	30.2	82%	206	0.9	86	2450	89	2.39	88	2.72	1.59	94%	25.1	60	154.5	M	25/10/2001	14
CF	1	9.0%	14	156	142	40	98	98	20.8	129	32	83%	158	1.2	102	3149	82	3.42	91	4.51	0.84	80%	25.5	78	175	M	06/01/1983	15
CF	18	-2.2%	-1	46	47	29.7	84	95	11.3	85	33	80%	152	0.87	63	1650	30	1.15	54	2.05	-0.83	20%	20.3	55.4	165	M	26/09/1998	16
CF	1	-10.3%	-6.6	64	70.6	46.07	97	98	10.6	105	30.6	96%	192	0.97	100	2027	73	1.6	90	2.27	1.37	91%	21.2	44	144	M	30/09/2005	17
CF	13	31.1%	24	77.2	53.2	44.3	98	97	9.6	107	26.6	91%	193	1.05	98	1750	80	1.93	88	2.45	0.12	55%	17.8	39.5	149	M	06/09/2004	18
CF	1	11.6%	5	43	38	40.2	100	100	5.9	104	35	85%	173	0.98	100	1008	65	1.08	94	1.64	-0.55	29%	15	25	129	F	30/05/2006	19
CF	3	0.0%	0	60	60	60.6	98	98	8.4	167	30	97%	195	1.09	149	1515	79	1.31	88	1.64	-0.017	43%	15.5	25	127	M	30/10/2006	20
CF	10	10.2%	10	98	88	46	99	99	11.5	97	37	98%	192	0.96	97	2379	86	2.47	90	3.01	1.04	85%	20.8	52	158	M	16/04/2002	21
CF	11	11.1%	5	45	40	46.8	94	96	6.5	113	41	89%	180	1	116	1133	64	1.13	74	1.38	-1.44	7%	13.9	24.2	132	F	22/06/2005	22
CF	20	-61.7%	-28	45.4	73.4	30.5	88	98	12.2	78	29.3	91%	172	0.91	72	2194	25	1.06	43	2.11	0.47	68%	24.1	72	173	M	28/01/1988	23
CF	16	16.9%	8.7	51.6	42.9	44.58	99	100	6.3	122	29.8	92%	185	1.14	113	1159	76	1.29	99	1.76	-0.83	20%	15.4	26	130	F	16/11/2006	24
CF	5	29.8%	34	114	80	42	96	96	15.2	108	28	78%	159	1.11	88	2450	85	2.85	89	3.47	0.11	54%	21	58	166	M	23/08/1997	25
CF		30.9%	29	94	65	42	100	97	8.8	110	30	100%	207	1.22	121	1904	97	2.28	100	2.52	0.72	76%	20.5	45	148	F	24/09/2001	26
CF	4	25.2%	39	155	116	48	98	98	15.3	99	26	94%	190	1.18	89	3027	102	3.9	106	4.72	0.73	77%	24	71	172	M	17/05/1996	27
CF	18	16.2%	16	99	83	36.2	98	100	8.9	96	31	89%		1.17	104	1630	86	2.2	103	2.85	1.1	86%	25.4	84	182	M	15/10/1996	28
CF	13	20.2%	7.6	37.6	30	22.9	84	94	7.5	60	29.2	81%	150	0.84	47	1121	24	0.94	46	2.1	-2.51	1%	17.4	49	168	M	18/07/1983	29
CF	5	42.0%	58	138	80	47	97	96	13.3	105	25	90%	180	1.29	93	2400	89	3.45	86	3.81	-1.19	12%	19.6	50.8	161	M	23/09/1994	30
CF	17	12.6%	8.9	70.9	62	27.1	98	98	9.4	74	38	74%	154	1.01	72	1349	51	1.4	66	1.99	-0.78	22%	19.5	49.2	159	F	05/05/1992	31
CF	16	27.1%	39.5	146	106.5	43.22	100	100	14.7	91	31.7	93%	179	1.02	86	2766	76	3.65	76	4.36	-1.44	7%	19.1	64	183	M	20/03/1995	32
CF	11	28.4%	19	67	48	52.3	100	100	6.9	137	31	89%	176	1.28	130	1308	106	1.6	126	2.01	-0.3	38%	16	25	125	F	01/05/2004	33
CF	11	15.0%	7.5	50	42.5	34.84	100	98	4.7	94	36.2	96%	194	1.21	86	871	72	1.25	69	1.35	-0.65	26%	14.8	25	130	M	15/07/2009	34
CF	13	24.5%	13	53	40	48	98	100	8	100	35	88%		1.1	114	1400	74	1.34	80	1.65	-0.26	40%	16.4	29	133	M	02/04/2003	35
CF		8.2%	9	110	101	43.6	98	100	14.7	100	30	83%	172	1.12	91	2528	76	2.75	77	3.26	0.14	56%	20.1	58	170	M	27/05/1999	36
CF	6	18.6%	24	129	105	50.9	99	99	14.5	107	25	96%	198	1.11	107	2850	99	3.24	93	3.55	0.27	61%	20.6	56	165	M	01/04/1999	37
CF	9	0.8%	1.2	142	140.8	53	98	96	22	123	32	90%	180	1.09	113	3966	68	2.86	116	5.61	-0.4	34%	21.4	74	186	M	23/06/1994	38
CF	10	15.3%	13	85	72	38	100	100	8.1	85	37	87%	170	1.05	107	1531	74	2.08	89	2.73	-1.71	4%	15.6	40	160	F	06/03/2000	39
CF		17.4%	21	121	100	57	99	99	13.2	93	30	98%	202	1.06	122	2782	102	3.53	102	4.11	-1.07	14%	17.4	49	168	M	06/03/1999	40
CF	10	43.4%	36	83	47	24.1	99	99	10.4	109	31	80%	149	1.06	86	1546	76	2.44	79	2.52	1.01	84%	26	64	157	F	16/04/1981	41
CF	3	33.8%	26	77	51	22.4	100	100	9.3	97	30	74%	143	1.18	73	1338	67	1.94	64	2.15	0.69	75%	24.2	59.7	157	F	15/04/1986	42
CF		6.6%	6	91	85	24.5	98	98	11.3	103	32	89%	176	1.14	93	2038	65	2.29	80	3.19	1.4	92%	28.7	82	169	F	16/09/1990	43
CF	12	24.1%	41	170	129	43.8	96	98	22	129	28	97%	169	1.07	125	3800	71	3.04	90	4.77	0.83	80%	25.4	88	186	M	06/05/1971	44
CF	4	32.9%	27	82	55	29	100	100	8.1	77	37	89%	172	0.93	66	1388	63	2.07	80	3.04	1.19	88%	27.1	72	163	F	01/01/2013	45
CF	10	46.3%	62	134	72	22.8	98	100	11.9	70	26.6	89%	167	1.22	58	1894	75	3.33	80	4.24	0.8	79%	25.3	83	181	M	28/02/1984	46
CF	10	2.8%	1.8	63.2	61.4	25.6	98	98	10.6	113	35	83%	171	0.99	79	1820	57	1.58	86	2.58	1.72	96%	28.1	71	159	F	07/01/2000	47
CF	9	-20.7%	-12	58	70	30	97	97	9.5	90	37	94%	190	1.09	91	1814	46	1.36	99	3.23	0.19	58%	21.8	60	166	F	18/01/1996	48
CF	3	29.3%	27	92	65	39	100	100	8.9	88	30	89%	186	0.96	83	1817	96	2.36	92	2.62	0.73	77%	20.9	47	150	M	23/09/2000	49
Bronchiolitis obliterans	6	1.6%	1	64	63	50	100	100	9.2	100	29	95%	188	1.11	114	1765	71	1.63	91	2.39	-1.01	16%	16.4	35	146	M	04/11/2001	50
s/p TE Fistula	2	12.5%	6	48	42	41.4	100	100	7.5	89	34	102%	193	1.04	85	1408	39	1.12	38	1.25	-4.6	1%	14.3	34	154	F	22/09/1988	51
Post infectious	7	51.6%	64	124	60	27	100	100	9.2	94	31	85%	176	1.15	84	1627	109	3.1	116	3.67	0.98	84%	22.1	58	162	F	24/04/2001	52
PCD	9	12.6%	6	47.6	41.6	21.6	77	86	10.8	72	25.5	84%	150	0.88	61	1617	30	1.14	49	2.24	0.67	75%	24.8	75	174	M	20/11/1972	53
Bronchiolitis obliterans	12	15.1%	8	53	45	23.5	100	100	6.8	72	37	94%	178	1.05	64	1200	44	1.33	54	1.87	-0.33	37%	20.6	52	159	F	26/01/1992	54
Bronchiolitis obliterans	11	-14.3%	-5	35	40	35.5	99	99	7.9	88	33	93%	183	0.85	78	1455	38	0.87	57	1.53	0.26	60%	19	41	147	M	18/11/2001	55
Bronchiolitis obliterans	18	42.3%	44	104	60	34	94	96	11.2	74	32	84%	163	1.08	72	1891	67	2.62	99	4.51	-1.61	5%	18.2	55	174	M	13/12/1995	56
Bronchiolitis obliterans	10	23.4%	11	47	36	45.4	100	100	6.7	116	30	89%	180	1.13	117	1203	67	1.17	78	1.56	-0.48	32%	15.4	26.5	131	M	27/09/2005	57
BPD	1	-25.0%	-8	32	40	28	98	97	7.5	80	29	90%	176	1.01	83	1413	40	1.06	60	1.73	0.69	75%	20.6	44.5	147	F	16/07/2001	58
BPD	3	45.1%	23	51	28	37	99	99	5.9	117	35	78%	154	0.9	91	925	76	1.27	73	1.38	-1.35	9%	15.3	25	128	M	05/09/2002	59
BPD	16	-6.9%	-2.4	34.6	37	39	81	96	6.1	95	40	82%	170	0.86	93	1048	37	0.68	51	1.06	-1.6	5%	15	27	134	M	07/12/2001	60
Post infectious	0	34.9%	30	86	56	24.3	99	99	11.7	77	27	89%	176	1.02	66	2052	71	2.14	76	2.65	2.32	99%	33	84.5	160	M	24/06/2000	61
Other	6	3.6%	2	56	54	45.7	99	100	8.7	79	30	90%	178	1.03	103	1600	53	1.41	56	1.71	-1.82	3%	14.8	35	154	M	19/01/2002	62
PCD	14	13.9%	5	36	31	4.05	100	100	5.4	109	35	91%	183	0.96	102	994	45	0.67	48	0.75	-0.44	33%	15.6	24	124	F	07/07/2005	63
PCD	11	-20.5%	-6.8	33.2	40	56.6	100	100	6.1	121	36	104%	208	0.9	146	1217	50	0.83	73	1.38	-2.43	1%	13.1	21.5	128	M	26/10/2006	64
PCD	12	45.8%	32.6	71.2	38.6	23.64	100	100	7	82	30.7	92%	174	1.06	61	993	72	1.78	69	1.96	-0.56	29%	22.3	42	145	F	06/02/1989	65
PCD	7	45.3%	55.4	122.4	67	28	99	99	9.5	93	30	93%	186	1.15	86	1767	93	3.06	94	3.51	0.67	75%	24.2	62.6	161	F	18/12/1994	66
PCD	9	34.3%	33.5	97.6	64.1	32.43	98	100	9.5	100	29.8	95%	186	1.08	96	1816	94	2.44	120	3.39	0.94	83%	22.3	56	155	F	17/05/2001	67
PCD		36.6%	37	101	64	36.6	98	100	9.6	98	33	93%	190	1.02	106	1818	90	2.54	102	3.19	0.5	31%	19.5	50.6	161	F	27/05/1997	68
PCD	15	1.0%	1	99	98	41.4	98	100	13.1	86	32	101%	195	1	85	2555	55	2.47	80	4.26	-1.25	11%	19.5	61.7	178	M	04/08/1992	69
PCD	12	31.7%	22.8	72	49.2	31.15	98	99	8.4	104	29.5	87%	171	1.09	90	1433	62	1.8	76	2.49	-1.24	11%	17.1	46	164	F	10/11/2001	70
PCD	5	-2.9%	-1	34	35	30	100	100	5.3	67	40	91%	191	0.96	80	1161	41	0.85	65	1.42	-0.72	24%	15.8	31	140	F	05/09/2002	71
PCD	19	1.2%	1	86	85	43	94	100	13.9	96	31	92%	184	1.02	86	2541	49	2.17	64	3.36	-1.84	3%	18.4	57	176	M	30/03/1991	72
PCD	11	34.3%	49	143	94	41.2	99	99	11.6	78	35	98%	191	1.1	83	2476	81	3.59	92	4.75	-0.07	47%	22.3	60	164	M	28/02/1994	73
Other	12	18.8%	19	101	82	38.7	100	100	10.2	75	35	98%	193	1.05	71	1975	70	2.53	79	3.32	-1.9	3%	17.6	51	170	M	10/03/1997	74
s/p TE Fistula	9	-8.8%	-5.2	58.8	64	30	99	99	9.5	101	38	91%	182	0.94	62	1750	63	1.47	74	1.99	2.18	98%	26.4	57	147	M	03/02/2004	75
s/p TE Fistula	3	35.5%	13.2	37.2	24	33.6	96	96	4.9	77	30	79%	164	0.99	83	807	49	0.93	55	1.09	-3.27	1%	13.2	24	135	F	26/08/2001	76
s/p TE Fistula	7	16.7%	12	72	60	40	100	100	9	91	30	91%	182	1.17	78	1642	52	1.74	62	2.33	-1.67	5%	18.7	41.5	149	M	26/10/1994	77
PCD	17	33.2%	25.2	75.8	50.6	17.6	98	97	10.9	119	31.1	88%	145	0.96	110	1584	72	1.6	79	2.09	1.9	97%	34.3	90	162	F	09/08/1951	78
Other	1	18.4%	10.8	58.8	48	37.7	100	100	7.3	94	36	87%	184	0.97	85	1346	72	1.47	94	2.2	1.09	86%	18.2	35.7	140	M	16/02/2006	79
Other	8	15.6%	12	77	65	36.55	100	100	8	87	35	89%	179	1.21	79	1462	72	1.92	67	1.95	-1.76	4%	16.2	40	157	F	03/08/1998	80
Other	10	23.6%	25	106	81	33.5	100	100	9.9	73	40	94%	184	1.07	66	1825	82	2.65	98	3.68	-0.6	27%	19.2	52.9	166	M	06/04/1998	81
Other	2	41.7%	28.7	68.8	40.1	53.73	98	98	6.9	94	27.9	91%	182	1.01	84	1252	106	1.72	112	2.07	-0.98	16%	22.3	23.3	126	M	05/07/2007	82
Idiopathic bronchiectasis	12	56.7%	59	104	45	17.4	100	100	9.4	87	37	77%	154	0.85	67	1442	79	2.6	80	3.02	1.69	95%	31.5	82.7	162	F	18/03/1994	83
Idiopathic bronchiectasis		-12.5%	-10	80	90	40.2	100	98	14.1	100	40	81%	157	0.96	78	2215	46	2.01	52	2.62	-1.79	4%	18.5	55	172.5	M	26/07/1994	84
Idiopathic bronchiectasis	12	30.0%	18	60	42	33.3	100	100	7.4	105	29	94%	190	1.02	96	1421	74	1.51	81	1.76	1.38	92%	21.4	42	140	F	21/10/2004	85
Idiopathic bronchiectasis	9	35.9%	37	103	66	25.3	100	100	7.6	53	35	91%	175	0.93	77	1600	72	2.59	77	3.16	-0.08	47%	21.3	63	172	F	12/01/1991	86
Idiopathic bronchiectasis	12	41.6%	32	77	45	22.7	100	100	5.1	57	31	94%	185	1.28	64	945	76	1.93	88	2.41	-1.49	7%	17.7	42	154	F	26/10/1996	87
Idiopathic bronchiectasis	7	2.6%	1.6	61.6	60	56.77	100	100	9.1	138	30.5	97%	194	1.06	132	1760	71	1.54	85	2.12	-0.98	16%	15.2	31	143	M	29/12/2004	88
Idiopathic bronchiectasis		24.1%	21.6	89.6	68	26.7	98	100	10.3	52	26.5	105%	207	0.99	54	2134	62	2.24	77	3.23	1.75	96%	27.7	80	170	M	28/03/2000	89
Post infectious	14	-0.8%	-0.7	87.3	88	40	97	97	14.9	96	30	98%	193	1.1	81	2550	46	2.09	57	3.02	-0.7	24%	20.7	64	176	M	08/07/1995	90
Post infectious	12	60.6%	67.6	111.6	44	25.4	98	98	10.8	113	23.2	94%	166	1.03	106	1799	97	2.79	118	3.94	0.95	83%	25.6	71	166.5	F	04/10/1970	91
Idiopathic bronchiectasis	8	38.0%	67.8	178.4	110.6	52.98	98	99	16.1	99	26.4	99%	194	1.13	98	3126	96	4.46	98	5.36	-2.04	2%	17.4	59	184	M	30/08/1998	92
Idiopathic bronchiectasis	1	18.5%	12	65	53	49	97	99	8.8	116	29	87%	184	1	112	1616	80	1.62	108	2.5	0.43	67%	17.1	33	139	M	12/06/2005	93
Idiopathic bronchiectasis	6	32.4%	28.8	88.8	60	32.06	98	97	7.7	88	33.7	102%	203	1	93	1571	86	2.22	106	2.95	0.56	71%	20.7	49	154	F	20/12/2001	94
Idiopathic bronchiectasis	8	-1.8%	-1	57	58	42.8	99	99	8.1	132	28	85%	170	1.14	116	1500	79	1.42	95	1.81	1.19	88%	19.8	35	133	F	27/10/2005	95
Post infectious	4	33.3%	29	87	58	44	99	97	10.2	106	30	83%	172	1	97	1800	92	2.18	99	2.7	0.35	64%	18.9	41.5	148	M	03/09/2001	96
PCD	18	19.4%	12	62	50	25	78	93	11.8	76	31	97%		1.1	74	1979	31	1.23	51	2.53	0.33	63%	23.5	78	182	M	05/05/1966	97
Idiopathic bronchiectasis	11	54.2%	78	144	66	35.7	99	99	13.7	93	34	87%	170	1.08	65	1965	85	3.6	81	4.02	-2.19	1%	17.2	55	179	M	21/09/1996	98
Idiopathic bronchiectasis		32.6%	59	180.8	121.8	46.8	99	99	17.9	111	26.4	95%	186	1.06	102	3324	119	4.52	106	4.73	0.65	74%	23.7	71	173	M	11/07/1997	99
Idiopathic bronchiectasis	19	-8.0%	-7.2	89.8	97	32	99	99	12.8	86	31	94%	180	1.1	76	2302	50	2.09	70	3.46	0.55	71%	24.3	72	172	M	16/09/1988	100
Post infectious	7	25.4%	41	161.6	120.6	51.84	100	100	16.7	101	26.8	99%	195	1.09	108	3266	97	4.04	104	5.1	-0.23	41%	19.9	63	178	M	31/03/1999	101
Post infectious	1	37.9%	39	103	64	27.4	98	100	11.5	98	24	98%	202	1.19	100	2324	92	2.58	100	3.28	2.44	99%	34.4	84.8	157	M	02/08/2001	102
